# Consensus Document of the Spanish Nutrition Society (SEÑ) on Nutritional Strategies in Sports

**DOI:** 10.3390/nu17243862

**Published:** 2025-12-11

**Authors:** Juan Mielgo-Ayuso, Adrián Macho-González, Natalia Úbeda, Antonio Jesús Sánchez-Oliver, María Martínez-Ferrán, Diego Fernández-Lázaro, Raquel Aparicio-Ugarriza, Enrique Roche, Marcela González-Gross

**Affiliations:** 1 Faculty of Health Sciences, University of Burgos (UBU), 09001 Burgos, Spain; 2 Advanced Research in Integrative Physiology for Life Research Group (IAFIV), University of Burgos (UBU), 09001 Burgos, Spain; 3 Working Group on Nutrition for Exercise and Sport, Spanish Nutrition Society (SEÑ), 28010 Madrid, Spain; amacho@ucm.es (A.M.-G.); diego.fernandez.lazaro@uva.es (D.F.-L.); eroche@umh.es (E.R.); 4 Nutrition and Food Science Department (Nutrition), Pharmacy School, Complutense University of Madrid, 28040 Madrid, Spain; 5 AFUSAN Research Group, Sanitary Research Institute of the San Carlos Clinical Hospital (IdISSC), 28040 Madrid, Spain; 6 Departamento de Ciencias Farmacéuticas y de la Salud, Facultad de Farmacia, Universidad San Pablo CEU, CEU Universities, Urbanización Montepríncipe, Boadilla del Monte, 28660 Madrid, Spain; 7 Departamento de Motricidad Humana y Rendimiento Deportivo, Universidad de Sevilla, 41013 Sevilla, Spain; 8 Area of Histology, Faculty of Health Sciences, University of Valladolid, 42004 Soria, Spain; 9 Neurobiology Research Group, University of Valladolid, 47005 Valladolid, Spain; 10 Facultad de Ciencias Jurídicas, Educación y Humanidades, Universidad Europea de Madrid, Campus de Villaviciosa de Odón, Calle Tajo s/n, Villaviciosa de Odón, 28670 Madrid, Spain; 11 ImFINE Research Group, Department of Health and Human Performance, Universidad Politécnica de Madrid, 28040 Madrid, Spain; 12 CIBER Fisiopatología de la Obesidad y Nutrición (CIBEROBN), Instituto de Salud Carlos III (ISCIII), 28029 Madrid, Spain; 13 Department of Applied Biology-Nutrition, Institute of Bioengineering, University Miguel Hernández, 03202 Elche, Spain; 14 Alicante Institute for Health and Biomedical Research (ISABIAL), 03010 Alicante, Spain; 15 Spanish Nutrition Society (SEÑ), 28010 Madrid, Spain

**Keywords:** sports nutrition, performance, dietary strategies, carbohydrate periodization, intermittent fasting, ketogenic diet, plant-based diet, consensus, guidelines

## Abstract

**Introduction**: Nutrition plays a fundamental role in sports performance by influencing energy availability, recovery, and training adaptation. In recent years, different dietary strategies have gained popularity among athletes, although the evidence supporting their efficacy is inconsistent. **Objective**: This consensus document, developed under the auspices of the Spanish Society of Nutrition, aims to provide a critical overview of the most relevant nutritional strategies currently used in sports and to offer evidence-based practical recommendations for both professional and recreational athletes, coaches, and health professionals. **Methods**: A narrative review was conducted following standardized scientific procedures by a multidisciplinary panel of experts. The analyzed strategies included high-carbohydrate, low-carbohydrate, ketogenic, intermittent fasting, plant-based, Paleolithic, and carbohydrate periodization diets. Each strategy was assessed based on its physiological rationale, evidence of performance in endurance, strength/power, sprint, aesthetic, weight category, and team sports, practical applications, and potential risks. **Results**: The available evidence shows that no single dietary strategy can be universally recommended for all athletes. High carbohydrate availability remains the most consistent approach for sustaining performance in endurance and high-intensity efforts. Low-carbohydrate and ketogenic diets enhance fat oxidation but often compromise exercise economy at competitive intensity levels. Intermittent fasting may improve body composition and metabolic health; however, it requires careful adaptation. Well-planned plant-based diets can support performance, although attention to certain nutrients (e.g., B12, iron, and omega-3) is essential. Paleolithic diets improve metabolic parameters but show limited direct evidence of athletic performance. Carbohydrate periodization is a promising tool for combining metabolic adaptations with competitive demands. **Conclusions**: Nutritional strategies should be individualized according to the athlete’s sport, training phase, and personal context. Professional guidance is crucial for minimizing risks and optimizing benefits. Further well-designed, long-term studies on athletes are needed to resolve the current controversies.

## 1. Introduction

In recent decades, the role of nutrition in sports has been established as an essential element for optimizing the physical performance, recovery, and overall health of athletes. Proper nutritional intervention not only facilitates the maintenance of favorable body composition but also plays a decisive role in maximizing training capacity, reducing the risk of injuries, improving recovery efficiency, and prolonging athletic careers [1,2,3].

Advances in scientific knowledge have enabled the development of specific dietary strategies designed to modulate metabolism, promote physiological adaptations, and enhance performance in various sports. Among these, high-carbohydrate diets (HCD), low-carbohydrate diets (LCD), ketogenic diets (KD), intermittent fasting (IF), plant-based diets (vegetarian and vegan diets- VEG), Paleolithic diets (PALEO), and carbohydrate periodization (CP) are noteworthy [3,4,5].

The popularization of simplified messages on social media and other mass communication channels has amplified the popularity of these nutritional approaches. In many cases, the shared recommendations lack a rigorous scientific background or are presented in a biased manner, prioritizing aesthetic results or quick promises over health and long-term performance [6]. This has encouraged the adoption of restrictive or unbalanced diets without proper professional supervision, with potential health risks, including deficiencies in essential nutrients, hormonal imbalances, decreased physical performance, and the onset of eating disorders [7,8].

Simultaneously, the science of sports nutrition has evolved toward an increasingly individualized approach. Personalizing dietary strategies based on the type of sport, timing within the season, training level, nutrient availability, and physiological and psychological characteristics of athletes is now recognized as a fundamental principle for achieving optimal results [9,10]. Furthermore, the influence of cultural, ethical, and environmental factors on food choices is becoming increasingly valued, particularly in the context of plant-based diets and sustainability [11].

**Figure 1:**
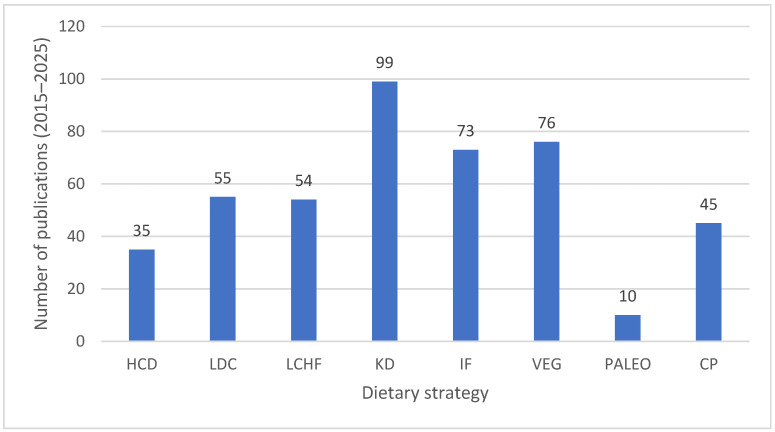
Scientific output on dietary strategies used in sports (PubMed results, 2015–2025). Data were derived from structured PubMed searches using standardized Boolean terms (“high-carbohydrate diet,” “low-carbohydrate diet,” “low-carbohydrate high-fat diet,” “ketogenic diet,” “intermittent fasting,” “vegetarian diet,” “vegan diet,” “Paleolithic diet,” “carbohydrate periodization”) combined with “athletes” AND “sports nutrition,” with a 10-year filter applied (2015–2025). Publication counts reflected the total number of indexed articles for each dietary strategy. Abbreviations: HCD, high-carbohydrate diet; LCD, low-carbohydrate diet; LCHF, low-carbohydrate, high-fat diet; KD, ketogenic diet; IF, intermittent fasting; VEG, vegetarian/vegan diet; PALEO, Paleolithic diet; CP, carbohydrate periodization.

Despite the growing interest and significant volume of research published in recent decades, considerable methodological and outcome heterogeneity remains among studies exploring the effects of these nutritional strategies on athletic performance. Differences in study design, intervention duration, participant level and characteristics, and methods used to assess performance make it challenging to extrapolate general conclusions and formulate universal recommendations [12,13]. To illustrate the uneven growth of research across dietary approaches, Figure 1 summarizes the number of publications indexed in PubMed over the last decade for the main strategies analyzed in this consensus document.

For example, although there is extensive evidence supporting the use of high-carb diets to optimize performance in endurance sports, in recent years, interest has grown in alternative strategies, such as LCD or KD, the effectiveness of which remains a subject of debate and shows contradictory results depending on the context and type of sports discipline [14,15].

IF has gained popularity as a strategy for improving body composition and metabolic health. However, its impact on athletic performance varies considerably depending on the duration and type of protocol, timing of food intake, and the discipline practiced [16]. Although the PALEO diet is based on an evolutionary approach and prioritizes minimally processed foods, its naturally low carbohydrate availability may limit its suitability for sports that predominantly rely on anaerobic–glycolytic metabolism (e.g., sprinting, middle-distance events, combat sports) [17].

Finally, nutritional periodization has emerged as an advanced strategy that proposes adapting nutrient intake, especially carbohydrates (CHO), to the training and competition cycle, aiming to maximize physiological adaptations without compromising performance or health [18]. This integrative approach is based on the idea that there is no single optimal strategy for all athletes and that flexibility and individualization are key to success.

In this context of great diversity and complexity, the need for a consensus document that is critically up to date, synthesizes the available evidence, and offers clear and practical guidelines is evident. The Spanish Society of Nutrition, aware of the central role of nutrition in health and sports performance, promotes this document as a tool to support informed and safe decisions by athletes, coaches, dietitian-nutritionists, and other health professionals.

The recommendations formulated throughout this consensus are intended to have a broad and adaptable scope, addressing the needs of elite athletes, recreational practitioners, and athletes in training. Although the scientific evidence is derived from studies conducted in populations with varying levels of performance, the objective is to provide a robust and practical framework that can be interpreted and applied by professionals responsible for nutrition planning in diverse sporting contexts. Accordingly, this document aims to serve as a flexible and useful reference to support health, performance, and nutritional safety across a wide range of athletic profiles.

This consensus document has the following objectives.

- To critically and comprehensively review the principal dietary strategies used in sports, the scientific foundations, physiological mechanisms, and contexts of their applications are described.
- To analyze the impact of each strategy on sports performance, its application in endurance sports, strength and power sports, sprint, aesthetic, weight-category, and team sports, while considering the specific characteristics of each type of sport.
- To provide practical and applicable recommendations based on the best available evidence for the safe and effective implementation of each strategy, highlighting the importance of individualization and professional supervision.
- To identify current controversies and methodological limitations and propose future lines of research that will strengthen the scientific foundation and improve both clinical and sports practice.

This review serves as a reference guide for athletes, coaches, dietitians, nutritionists, and health professionals, encouraging informed decision-making, avoiding the adoption of unsupported or trend-based strategies, and contributing to the promotion of health and optimal performance.

## 2. Dietary Strategies Used in Sports

Before examining each dietary strategy in detail, Table 1 provides an updated comparative synthesis of their characteristics. It summarizes:

(i) the recommended context of application,
(ii) potential benefits,
(iii) limitations or risks, and
(iv) key practical recommendations.

**Table 1:** Summary of main dietary strategies used in sports.

Strategy (Abbreviation)	Recommended Context	Potential Benefits	Limitations/Risks	Key Recommendations
High-carbohydrate diet (HCD)	High glycolytic demand; rapid recovery needs; prolonged/intense sessions. Common in endurance and team sports.	Maximizes glycogen stores; supports high-intensity performance and recovery.	Excess caloric intake, if poorly adjusted, may be unnecessary for low-intensity training.	Adjust intake to session volume and intensity; emphasize timing.
Low-carbohydrate diet (LCD)	Aerobic base training; metabolic-flexibility goals; fat-loss phases; ultra-endurance preparation.	Increases fat oxidation and metabolic flexibility.	Reduced anaerobic performance; micronutrient-deficiency risk.	Use short-term; avoid in high-intensity phases; monitor adaptation.
Low-carbohydrate high-fat diet (LCHF)	Same contexts as LCD, but with higher fat intake and more pronounced metabolic shifting.	Greater reliance on fat oxidation; possible endurance benefits in specific contexts.	Poor tolerance at high intensities; adherence challenges; potential nutrient deficiencies.	Implement with monitoring; assess training demands carefully.
Ketogenic diet (KD)	Weight reduction; specific metabolic management; selected preparatory phases.	Promotes fat oxidation; decreases fat mass; may reduce inflammation.	Impairs high-intensity performance; gastrointestinal issues; nutrient deficits.	Apply short cycles; require supervision; track biomarkers.
Intermittent fasting (IF)	Body recomposition; metabolic-health improvement; recreational athletes; off-season phases.	Fat loss; improved metabolic markers; enhanced insulin sensitivity.	Risk of lean-mass loss; reduced tolerance to heavy training loads.	Individualize; avoid during competition periods or very intense training.
Vegetarian/Vegan diet (VEG)	Cardiovascular health; ethical/sustainability motivations; structured long-term planning.	Compatible with performance; lower cardiovascular risk.	Risk of B12, iron, and omega-3 deficiencies; lower energy density; sustainability varies.	Ensure complete proteins; monitor critical nutrients; supplement when needed.
Paleolithic diet (PALEO)	Body-composition improvement; glycemic management; off-season phases.	Nutrient-dense foods; supports glycemic control.	Restricted carbohydrate intake; potential calcium and vitamin D inadequacy.	Avoid during high-intensity cycles; individualize planning.
Carbohydrate periodization (CP)	Enhancing metabolic adaptations by modulating carbohydrate availability across sessions.	Improves metabolic efficiency and substrate flexibility.	Complex application; risk of excessive fatigue if misapplied.	Use in selected sessions (“train-low”); alternate with high-availability days.

**Note**: The information summarized in this table is supported by the references cited in Section 2 (HCD: [1,5,19,20,21,22,23,24,25,26,27]; LCD/LCHF/KD: [12,28,29,30,31,32,33,34,35,36,37,38,39,40,41,42,43,44,45,46,47,48,49,50,51]; IF: [52,53,54,55,56,57,58,59,60,61,62,63,64]; VEG: [65,66,67,68,69,70,71,72,73,74,75]; PALEO: [76,77,78,79,80,81,82,83,84,85,86]; CP: [6,9,10,18,27,87,88,89,90]).

This overview is intended to serve as a conceptual guide and facilitate the interpretation of each approach before the detailed subsections.

The evidence presented in Table 1 is derived from major consensus statements, systematic reviews, controlled trials, and applied practice frameworks across sports nutrition.

- High-carbohydrate diets (HCD) and carbohydrate intake during exercise are supported by position stands and foundational applied work [1,5,19,20,21,22,23,24,25,26,27].
- Recommendations for low-carbohydrate diets (LCD), low-carbohydrate high-fat (LCHF) approaches, and ketogenic diets (KD) are derived from controlled trials, meta-analyses, and narrative reviews [12,28,29,30,31,32,33,34,35,36,37,38,39,40,41,42,43,44,45,46,47,48,49,50,51].
- Evidence for intermittent fasting (IF) comes from experimental trials and integrative reviews [52,53,54,55,56,57,58,59,60,61,62,63,64].
- Athlete-specific considerations regarding vegetarian/vegan (VEG) and predominantly plant-based dietary patterns are based on umbrella reviews, systematic reviews, and position statements [65,66,67,68,69,70,71,72,73,74,75].
- Findings related to Paleolithic diets (PALEO) are supported by classical and contemporary analyses [76,77,78,79,80,81,82,83,84,85,86].
- Carbohydrate periodization (CP) and strategies such as “train-low” or “sleep-low” are grounded in mechanistic models and intervention studies [6,9,10,18,27,87,88,89,90].

This unified summary framework supports the detailed evaluation of each nutritional strategy, as presented in the following subsections.

### 2.1. High-Carbohydrate Diets (HCD)

#### 2.1.1. General Description and Scientific Foundations

HCD represents the traditional and most evidence-based method for optimizing athletic performance, especially in endurance sports, team sports, and disciplines involving repeated high-intensity efforts. The choice of this nutritional approach is based on the ability of CHO to serve as the main energy substrate during moderate-to high-intensity activities [1].

CHO is stored as glycogen in the skeletal muscle (approximately 300–700 g) and liver (80–100 g), serving as an essential fuel to maintain exercise intensity and delay the onset of fatigue. When glycogen stores are depleted, there is a marked decline in performance, reduced strength, and an increased perception of effort [91].

From a molecular perspective, CHO availability influences various signaling pathways, such as mTOR and AMPK, modulating both protein synthesis and mitochondrial oxidative efficiency [90]. Moreover, an adequate supply of CHO helps maintain immune function and integrity of the central nervous system during prolonged exertion [92].

In practice, HCD typically provides between 50% and 65% of the total caloric intake, although the intake can exceed 70% during loading phases or periods of high-energy demand [5].

#### 2.1.2. Impact on Athletic Performance

##### Endurance Sports

The importance of CHO in endurance sports has been widely demonstrated. Adequate storage of muscle and liver glycogen allows athletes to maintain high intensity for prolonged periods and significantly delays fatigue. CHO-loading strategies before long competitions (carbo-loading) can increase muscle reserves by up to 50%, improving performance in events lasting > 90 min [25].

During exercise, the intake of exogenous CHOs maintains plasma glucose availability, preserves muscle glycogen, and helps support brain function, reducing perceived effort and the risk of “bonking” or “hitting the wall” [19]. For efforts lasting between 1 and 2.5 h, CHO intakes of 30–60 g/h are recommended [1]; however, in longer-duration events, such as marathons or ultra-trails, it has been shown that consuming between 90 and 120 g/h maximizes CHO oxidation, improves performance, reduces exercise-induced muscle damage, and speeds up the recovery of sprint capacity and neuromuscular function [21,22,23]. However, it is essential to train the digestive system (“gut training”) to avoid gastrointestinal discomfort, which can compromise the performance and tolerance of the nutritional plan [24].

##### Strength and Power Sports

Although CHO demands in strength and power sports are not as high as those in endurance sports, muscle glycogen is essential for repeated explosive efforts and maintaining training quality [93]. Adequate CHO availability, glycogen resynthesis between sessions, improves work capacity, and can enhance strength and muscle hypertrophy by enabling training with higher volumes and intensities [2].

##### Team Sports

Team sports are characterized by intermittent high-intensity efforts, alternating with incomplete recovery periods. In this context, muscle glycogen is crucial for sustaining sprint capacity, agility, and cognitive performance throughout the match [7].

CHO consumption before and during the match helps maintain optimal glucose levels and delays fatigue, especially in the final stages of the competition. Intakes of 30–60 g/h during the match have been shown to be effective for maintaining technical and tactical performance [7,94].

#### Sprint Sports

Sprint sports (track sprints, short-distance cycling, sled pushes) rely predominantly on the ATP–phosphocreatine (ATP–PCr) system and anaerobic glycolysis, making glycogen availability a key determinant of maximal force production and repeated sprint ability. High-CHO availability supports peak power output, neuromuscular firing rates, and buffering capacity during repeated maximal efforts [2,5,93,95]. Brain glucose availability also influences central drive and motor unit recruitment during very high-intensity exercise, reinforcing the importance of sufficient pre-training and pre-competition CHO intake [19]. In practice, CHO-rich pre-event meals and intra-session CHO (e.g., between sprint heats) help maintain performance and delay neuromuscular fatigue.

#### Aesthetic Sports

Aesthetic sports (artistic gymnastics, figure skating, dance) combine high technical skills, substantial training volumes, and chronic pressure toward low body mass, which increases the risk of low energy availability (LEA), menstrual dysfunction, and bone injury [96,97,98]. Adequate CHO intake is essential for sustaining training quality, concentration, technical skill acquisition, and recovery, particularly during long technical sessions in which glycogen depletion can compromise coordination and precision. HCD also reduces injury risk by supporting hormonal balance, immune function, and appropriate bone turnover—areas that are especially vulnerable in aesthetic athletes [96,97,98,99,100].

#### Weight-Category Sports

Weight-category sports (combat sports, lightweight rowing, and weightlifting) frequently require acute or chronic body mass manipulation, and athletes often restrict CHO intake as a rapid weight-cutting strategy. However, inappropriate CHO restriction can impair training quality and competition performance and increase the risk of relative energy deficiency in sports (RED-S) [96,99,100]. Strategically applied HCD, such as CHO tapering followed by reloading close to competition, helps maintain strength, speed, and hydration status while allowing regulated weight reduction [9,18,27]. Preserving glycogen availability is also crucial for repeated high-intensity actions during sparring or match simulations; thus, CHO intake should be modulated rather than eliminated during weight management phases.

#### 2.1.3. Diet-Specific Limitations

Although HCD is broadly supported, several limitations must be considered when applying it across different athletic contexts. Excessive reliance on rapidly absorbed CHO or large pre-exercise loads may increase the likelihood of gastrointestinal distress, particularly in athletes who have not undergone systematic gut-training protocols [24]. In weight-sensitive disciplines, sustained high-CHO availability may complicate body-mass regulation and predispose athletes to LEA episodes later in the season if caloric intake is not carefully coordinated with training load [96,97,98,99].

Chronic high-CHO intake without appropriate periodization can also limit metabolic flexibility during preparatory phases, reduce the ability to oxidize fats, or impair adaptations targeted through low-CHO availability models [12,90]. In sprint-dominant or highly technical sports, misalignment between CHO timing and session demands may result in unnecessary energy fluctuations or suboptimal neuromuscular readiness [2,5,93]. Finally, although HCDs are highly evidence-based for endurance and team-sport demands, their application to anaerobic, acyclic, or weight-dependent disciplines should be individualized to training structure, technical demands, and body-mass considerations [93,95].

#### 2.1.4. Practical Recommendations

##### Daily Intake of CHO

CHO intake planning should be tailored to the type of sport, seasonal phase, and training load. On a daily basis, recommendations vary according to the volume and intensity of the exercise. For light or technical sessions, consumption of 3–5 g of CHO per kg of body weight per day is suggested, while moderate-intensity training (1–2 h/day) requires between 5 and 7 g/kg/day. For prolonged or high-intensity training sessions (2–3 h/day), requirements may increase to 7–10 g/kg/day, and during specific loading phases before long competitions, an intake of up to 12 g/kg/day may be achieved [25].

For tests lasting longer than 90 min, it is recommended to carry out a CHO load involving an intake of 8–12 g/kg/day for 36–48 h prior to the event, accompanied by a progressive reduction in training volume and intensity to maximize muscle glycogen availability [25].

##### Intake During Exercise

During exercise, CHO needs depend on the duration of the effort. For activities lasting less than one hour, exogenous intake is usually not necessary. However, when exercise lasts between 1 and 2.5 h, it is recommended to consume 30–60 g of CHO per hour. For efforts longer than 2.5 h, intake can reach 90–120 g/h if combinations of different types of CHO (such as glucose and fructose) are used to increase the rate of exogenous carbohydrate oxidation and reduce gastrointestinal discomfort [21,22,23].

##### Recovery

The recovery phase after physical exercise is also important, especially when performing double sessions or intense workouts on consecutive days. In these cases, it is recommended to consume 1–1.2 g/kg/h of CHO during the first 4 h after exercise. Additionally, combining this with proteins (approximately 0.3 g/kg) has been shown to enhance protein synthesis and accelerate muscle recovery [101].

##### Practical Aspects

From a practical standpoint, it is essential to train the gastrointestinal system to tolerate HCD during exercise, a strategy known as gut training, which should begin with moderate doses (30–60 g/h) and be progressively increased. The choice of CHO source should be adapted to individual preferences and tolerances, and gels, isotonic drinks, bars, or other combined forms should be used. Similarly, it is recommended to integrate CHO intake with adequate hydration to facilitate absorption and prevent dehydration.

#### 2.1.5. Considerations

Excess CHO, without adjustment for energy expenditure, may lead to unwanted weight gain. Likewise, it is advisable to prioritize quality CHO (whole grains, fruits, and tubers) outside of competitions, reserving fast-acting CHO (high glycemic index) before, during, and after exercise [94].

### 2.2. Low-Carbohydrate Diets (LCD)

#### 2.2.1. General Description and Scientific Principles

LCDs are characterized by a significant reduction in CHO intake, generally below 20–25% of the total energy intake, or even less than 50 g/day in the most restrictive versions. The main goal of this strategy is to induce metabolic adaptations that enhance fat oxidation and minimize dependence on muscle and liver glycogen as the primary source of energy during exercise [102].

The key physiological mechanism involves the modulation of signaling pathways involved in mitochondrial biogenesis, such as the activation of AMPK, p38 MAPK, and p53. The combination of these signals increases the expression of cofactors, such as PGC-1α and mitochondrial proteins, promoting the muscle’s efficiency in using lipids as fuel. Likewise, the LCD increases the expression of fatty acid transporters (CD36) and carnitine palmitoyltransferase 1, facilitating the uptake of lipids at the muscular level and their subsequent oxidation [47,103].

Another relevant point is the increase in intramuscular triglycerides (IMTG), which is observed at rest and can reach an increase of 50 to 123% after several weeks of adaptation. These stores act as immediate sources of energy during exercise, especially at submaximal intensities [47].

Historically, the dominant paradigm holds that CHO are essential for performance because of the critical role of glycogen as an energy substrate in high-intensity and endurance sports [26]. However, starting in the 1980s and 1990s, LCD began to be explored to improve the efficiency of fat oxidation and promote metabolic flexibility [104]. This interest has been reinforced over the past decade by the search for methods to optimize body composition and modulate adaptive signaling for training.

#### 2.2.2. Impact on Athletic Performance

##### Endurance Sports

Ultra-endurance athletes adapted to LCD for prolonged periods (>9 months) have been shown to maintain a capacity for glycogen storage and utilization similar to that observed in athletes on HCD, while their maximum rates of fat oxidation can double [14]. These results have led to the consideration of the LCD as a potential option for ultra-endurance sports in which glycogen preservation is critical.

However, it has been reported that this increase in lipid-dependence increases the energy cost of exercise and reduces mechanical efficiency, which can negatively impact events that require high submaximal intensities or decisive final sprints [12,44].

In recreational runners and cyclists, some studies have not observed significant differences in submaximal performance after LCD; however, benefits have been reported in the reduction in fat mass and glycemic control parameters [31,32]. These findings suggest possible usefulness during the pre-season phases or periods focused on improving body composition. In contrast, in elite runners, the LCD has shown negative effects on running economy and performance during competitive events, confirming that adequate CHO intake remains crucial in situations of high glycolytic demand [33,44].

##### Strength and Power Sports

Performance in strength and power sports largely depends on the availability of muscle glycogen, which is essential for brief and explosive effort. LCD can limit the ability to perform multiple high-intensity repetitions and decrease power output [34,35].

Some studies have indicated that, although no significant decrease in maximum strength is observed if protein intake is adequate, the ability to maintain performance over repeated sessions may be compromised [36].

In athletes who need to rapidly reduce body weight, the LCD can be useful, as long as protein intake (≥1.6 g/kg/day) is maintained and it is strategically planned [37,38].

##### Team Sports

Team sports combine intermittent effort with rapid and repeated high-intensity transitions. In this context, adequate CHO availability is critical for sustaining sprint capacity and recovery between actions [39].

LCD in basketball and football players has shown decreases in the total work performed, as well as alterations in anabolic hormones and bone remodeling parameters. This can affect competitive performance and increase the risk of injuries and long-term bone health issues, particularly in young and developing populations [32,38].

##### Sprint Sports

In sprint sports, maximal power output relies heavily on phosphocreatine and fast glycolysis. The reduced glycogen availability typically induced by LCD can compromise repeated sprint ability, neuromuscular drive, and total work performed [34,35,39,93]. LCD may be useful only during short body-composition phases, during high-intensity training blocks. However, sufficient CHO availability is essential to maintain peak power and technical execution [36,37,38]. Therefore, LCD in sprinters should be temporary, carefully controlled, and never coincide with key intensity sessions.

##### Aesthetic Sports

Aesthetic athletes often face chronic pressure to maintain low body mass, which increases the risk of LEA, menstrual dysfunction, and bone-turnover disturbances [96,97,98]. Because LCD tends to reduce total energy intake and limit glycogen availability, it may impair training quality, technical skill acquisition, and recovery during long technical sessions. Their restrictive nature can exacerbate LEA and RED-S risk, making LCD generally unsuitable in aesthetic sports, except possibly in brief, closely monitored off-season phases.

##### Weight-Category Sports

Athletes in weight-category sports frequently use LCD for rapid weight manipulation. Although such approaches can produce short-term reductions in body mass, they may simultaneously impair high-intensity performance, hydration, and recovery [37,38,99]. Glycogen depletion reduces strength, power, and repeated-effort capacity—key components in combat sports and lightweight rowing. If used, low-CHO phases should be brief, medically supervised, and followed by controlled CHO reintroduction before competition.

#### 2.2.3. Diet-Specific Limitations

The available scientific evidence shows great heterogeneity, with small sample sizes, variability in protocols, and few long-term controlled studies [40]. Moreover, there is significant individual variability in the response to CHO restriction, which underscores the need for individualized assessment before its implementation [41].

#### 2.2.4. Practical Recommendations

The implementation of LCD should be based on clear objectives and be strictly supervised by a specialized professional. This strategy should be considered during specific phases, such as the pre-season, to promote adaptations in fat oxidation and improve metabolic flexibility, while avoiding its application during competition phases or high-intensity sessions [33,44,102].

In endurance sports, it can be incorporated as a “train low” strategy, always combined with sessions of high CHO availability to maintain performance capacity during key efforts [44,90].

In strength and power sports, an adequate protein intake (1.6–2.2 g/kg/day) must be ensured in strength and power sports, and performance and recovery should be monitored closely. LCD can be applied temporarily during periods of weight loss, avoiding compromise of lean mass [36,38].

In team sports, the LCD should be used with caution, considering its potential negative impact on intermittent performance and recovery, which are fundamental elements in these disciplines [39].

Additionally, it is essential to monitor the intake of critical micronutrients, such as calcium, magnesium, and potassium, which may be reduced in LCD and affect neuromuscular function and overall health [42,89]. Therefore, it is recommended to consider supplementation or careful selection of foods rich in these minerals.

Adapting to the LCD can be more challenging in hot environments because of changes in thirst sensation and water retention; therefore, hydration and temperature control strategies should be strictly enforced [40].

Finally, it is essential to carry out individualized follow-up, assessing parameters such as body composition, biochemical and hormonal responses, and subjective perception of performance. Thus, the strategy can be adjusted to optimize the results and minimize risks by dynamically adapting it to the athlete’s needs and objectives.

### 2.3. Ketogenic Diet (KD)

#### 2.3.1. Overview and Scientific Principles

The KD is characterized by a drastic restriction of CHO intake, generally less than 50 g/day or 5–10% of total caloric intake, along with a high consumption of fats (70–80%) and a moderate intake of proteins (approximately 0.8–1.0 g/kg of body weight). This distribution aims to induce a metabolic state known as nutritional ketosis, in which the body uses ketone bodies (acetoacetate, β-hydroxybutyrate, and acetone) as its main source of energy instead of glucose [105].

The main mechanism is the depletion of glycogen stores and the consequent decrease in oxaloacetate availability necessary for the Krebs cycle. This deficit promotes the accumulation of acetyl-CoA and its conversion to ketone bodies in the liver. These ketone bodies are subsequently used by the brain, skeletal muscle, and other tissues as alternative energy sources [106].

Unlike diabetic ketoacidosis, nutritional ketosis induced by a KD is a controlled physiological state, with serum ketone body concentrations generally between 0.5 and 3 mM. This state has been associated with improvements in insulin sensitivity, reduced fat mass, and beneficial changes in some cardiometabolic markers [30].

KD was initially developed as a therapy for controlling drug-resistant epilepsy in children. In recent decades, it has gained popularity among the general population and athletes because of its potential effects on body composition and metabolic flexibility [29].

#### 2.3.2. Impact on Athletic Performance

##### Endurance Sports

The KD has been shown to significantly increase fat oxidation during submaximal exercise (~1.5 g/min), even in trained athletes, which could be useful in very long-duration events in which glycogen preservation is crucial [29,48,50,51]. However, studies on elite athletes have shown a decrease in performance, especially at submaximal and high intensities, owing to the increased energy cost associated with using fat as the main source of energy and the lower availability of muscle glycogen [28].

A recent systematic review concluded that although improvements in fat oxidation are observed, there is no clear evidence of benefits in oxidative performance, and in some cases, negative effects have been documented. Furthermore, most studies reporting these negative effects lasted less than six weeks, and it is unknown whether longer adaptation periods could modify these results [28,48,49].

##### Strength and Power Sports

KD tends to have neutral or even negative effects on the maximum strength and strength gains compared to diets with higher CHO content. CHO restriction reduces the ability to replenish glycogen, compromising performance in short and explosive efforts that depend primarily on this substrate [28,107].

A systematic review showed that, in strength and power training, the KD did not provide significant advantages, and several studies observed reductions in power and anaerobic capacity [49,107].

However, some individuals may tolerate the diet without major changes, highlighting the importance of individual variability and the need for a personalized approach [28].

##### Team Sports

In team sports, such as soccer or basketball, where intermittent high-intensity efforts are combined with periods of incomplete recovery, the KD may not be optimal. A study on semi-professional soccer players showed that after 30 days on a KD, the players reduced their fat mass without affecting their muscle mass, strength, or power. However, specific performance in competition has not been evaluated, and maintaining lean mass does not necessarily imply the preservation of anaerobic capacity or repeated sprint ability [43].

The reduction in glycogen levels could affect the ability to perform repeated high-intensity efforts, a fundamental characteristic of team sports. Therefore, although it may be useful for rapid weight loss, its application during competitive phases should be approached with caution and tailored to individuals.

##### Sprint Sports

KD significantly reduces glycolytic flux and glycogen availability, both of which are essential for maximal power and repeated sprints. Evidence consistently shows impairments in high-intensity exercise economy, anaerobic capacity, and power production under ketogenic conditions [28,49,50,51,93]. Although KD increases fat oxidation, this adaptation has no ergogenic value for sprint events. Consequently, KD is not recommended for competitive sprint athletes.

##### Aesthetic Sports

KD may facilitate short-term reductions in fat mass, but its restrictive nature increases the risk of LEA, micronutrient deficiencies, and hormonal disturbances in aesthetic sports, where RED-S prevalence is already elevated [96,97,98,99,100]. Reduced glycogen availability may compromise movement precision, explosiveness, and overall training quality. KD should therefore only be considered in very short, supervised blocks for body-composition management and not during technical or competitive periods.

##### Weight-Category Sports

KD is sometimes adopted to induce rapid weight loss, but the associated glycogen depletion reduces hydration status and total training capacity, increasing the risk of performance decrements in combat sports and rowing [28,43,49]. Reduced anaerobic output is particularly detrimental during decisive high-intensity exchanges. Although some athletes tolerate KD without large losses in lean mass, structured CHO refeeding before competition is essential to minimize negative impacts on performance.

#### 2.3.3. Diet-Specific Limitations

There are several methodological limitations in studies on KD and performance. Most interventions are short-term, with small sample sizes, and without adequate control of training status. Furthermore, the extent of adaptation to the diet varies among individuals and depends on the type of sports, intensity, duration, and competitive context.

On the other hand, the KD is not synonymous with being entirely “CHO-free.” In practice, some protocols include small, strategic amounts of CHO during training sessions, which could alter the impact on performance [30].

Adherence and acceptance are also significant challenges, particularly for athletes with high energy demands.

#### 2.3.4. Practical Recommendations

The KD can be considered a tool in specific contexts, but it should not be universally implemented. Its use can be evaluated in endurance sports with low-intensity demands or during pre-season phases, in which the main goal is to reduce body fat mass and improve metabolic flexibility [29,43].

Short-term application (2–3 weeks) is recommended during the pre-season, accompanied by training at moderate intensity and without major changes in pace. Subsequently, a higher-CHO diet can be reintroduced to optimize performance in more demanding sessions and competitions [30].

In strength, power, and team sports, a KD should be applied with great caution because of the possible decrease in performance during explosive and repeated efforts. If used, it is essential to ensure adequate protein intake (≥1.6 g/kg/day) to preserve the muscle mass and optimize recovery [107].

During implementation, parameters such as body weight, body composition, physical performance, subjective fatigue sensation, and recovery should be monitored. In addition, it is recommended to monitor biochemical markers such as ketone levels, lipid profiles, and renal function. Finally, nutritional education and individualization are fundamental to ensuring adherence and minimizing potential risks. The KD can be effective for certain athlete profiles, but it should always be applied under professional supervision and tailored to the needs, sports, and competitive schedule of each athlete.

### 2.4. Intermittent Fasting (IF)

#### 2.4.1. General Description and Scientific Foundations

IF is a dietary strategy characterized by alternating periods of normal food intake, with periods of total or partial restriction of caloric foods and beverages, except for water. There are several IF modalities, with the most common being alternate-day fasting (1:1 or 2:1 protocols) and time-restricted feeding (TRF), such as the 16:8 protocol, where fasting is performed for 16 h and food intake is concentrated within an 8-h window [53,108].

IF has become popular in recent years owing to its potential benefits for overall health, body composition, and longevity. On a metabolic level, prolonged fasting periods support weight loss by reducing fat mass, lowering blood pressure, and improving cardiometabolic parameters, such as insulin sensitivity and lipid profile [54].

Among the physiological mechanisms involved, autophagy activation is a cellular recycling process that contributes to the elimination of damaged components and metabolic adaptation. Additionally, IF stimulates pathways such as AMPK and sirtuin-1, which promote mitochondrial biogenesis and fatty acid oxidation, favoring greater energy efficiency and adaptation to prolonged exertion [109].

IF also modulates the expression of genes related to energy metabolism, oxidative stress, and inflammation, potentially providing additional health and physical benefits [54].

#### 2.4.2. Impact on Athletic Performance

##### Endurance Sports

IF, especially in its TRF form, appears to have positive effects on endurance sports. Improvements have been observed in VO_2max_, cardiac output, and muscle oxidative capacity, which translate into greater utilization of fatty acids as fuel and preservation of glycogen reserves [55,62].

The activation of AMPK and regulation of sirtuin-1 induced by fasting promote muscle adaptations that optimize metabolic efficiency and aerobic capacity [16,54]. Similarly, IF is associated with a reduction in systemic inflammation, an improvement in the power-to-body weight ratio, and a decrease in total fat mass, which are key factors in endurance sports [16].

A study of elite cyclists showed benefits in body composition and performance parameters after an 8-week TRF protocol without compromising strength or power [16].

##### Strength and Power Sports

In high-intensity disciplines that rely primarily on CHO availability, IF has shown mixed results. Some studies have documented a decrease in speed and power attributed to lower glycogen availability and a possible reduction in protein synthesis during periods of energy restriction [56,63,64].

However, other studies did not report significant changes or even showed that performance was not negatively affected after a minimum adaptation period (at least four consecutive days) [57,58,64]. This suggests the existence of an individual adaptation phase, in which the body adjusts to a new energy pattern.

It is important to note that most available studies focus on short-term interventions, so there is no conclusive data on the long-term impact of IF on strength and power performance [64].

##### Team Sports

The effect of IF on team sports, which are characterized by intermittent and high-intensity efforts, has been less studied and presents mixed results. Part of the controversy comes from the extrapolation of studies conducted during Ramadan, where abstinence from fluids is also imposed in addition to dietary restrictions. During Ramadan, a decrease in physical performance was observed, which was mainly attributed to dehydration and disruption of circadian rhythms [59].

Although the definition of IF and fasting during Ramadan may seem similar, in practice, they differ in key aspects, such as the possibility of drinking water and the distribution of meals, so the results are not directly comparable.

##### Sprint Sports

Sprint athletes depend on maximal glycolytic output, which can be compromised by fasted states that reduce glycogen availability and impair power production [56,63]. IF has been associated with decreased training intensity and neuromuscular output in high-intensity sessions unless feeding windows are carefully timed [57,63,64]. Therefore, IF is generally unsuitable on days involving speed development, sprint mechanics, or explosive power work.

##### Aesthetic Sports

In aesthetic sports, IF increases the risk of LEA, disrupted menstrual function, hormonal dysregulation, and impaired bone turnover—issues that are already prevalent in this population [96,97,98]. Prolonged fasting may also impair concentration, fine motor control, and technical skill acquisition during long rehearsals. IF should be considered only in highly controlled situations, with strict monitoring of energy availability, micronutrient intake, and recovery.

##### Weight-Category Sports

IF is sometimes used to facilitate weight reduction in weight-category sports, but extended fasting windows may compromise training intensity and recovery during combat-sport preparations or rowing [59,61]. Its catabolic potential increases the risk of lean-mass loss and reduced power output—both detrimental in these disciplines. If implemented at all, IF should be scheduled early in the preparation period and discontinued close to competition to preserve high-intensity performance.

#### 2.4.3. Diet-Specific Limitations

IF shows significant inter-individual variability in terms of adaptation and tolerance, making it difficult to extrapolate the results. In addition, most studies have been conducted under laboratory conditions and in recreational populations, limiting their practical application to competitive and elite contexts [64].

The lack of longitudinal studies and high-quality methodological research on IF in athletes prevents drawing definitive conclusions. Individualization and professional monitoring are essential for evaluating the response and adapting the strategy according to the athlete’s goals and characteristics.

#### 2.4.4. Practical Recommendations

IF can be a useful tool for athletes seeking to reduce fat mass or improve metabolic efficiency, as long as it is carried out progressively, individualized, and under professional supervision [57].

Among the different modalities, TRF is considered the option with the highest adherence and the lowest risk of compromising muscle mass and performance. It is recommended to start with shorter fasting periods (12 h) and gradually increase them to 16–18 h, adjusted according to individual response and tolerance [54].

It is advisable to have main meals during the day, aligned with circadian rhythms, to minimize the negative impacts on hormonal regulation and sleep quality [60]. Similarly, during the feeding window, it is important to ensure an adequate intake of calories and essential nutrients to support training and recovery.

In high-intensity sports or competitive phases, the implementation of IF should be carefully evaluated, prioritizing strategies that ensure CHO availability and optimize protein synthesis [61,63].

It is recommended to avoid prolonged fasting during sessions that require high glycolytic demand or explosiveness, as this could compromise performance and increase the risk of injury. Finally, it is crucial to monitor clinical markers (body composition, biochemical profile, micronutrient status, energy levels, and sleep quality) and adjust strategies according to the athlete’s progress and goals.

### 2.5. Diets Based on Plant Foods (VEG)

#### 2.5.1. General Description and Scientific Foundations

VEG are defined as eating patterns that exclude meat, fish, and their derivatives. Depending on the degree of restriction, they may include dairy and/or eggs (lacto-vegetarian, ovo-vegetarian, or lacto-ovo vegetarian diets) or exclude all animal products entirely (vegan diets) [65].

Interest in VEG has grown significantly for ethical, environmental, and health reasons [67]. From a public health perspective, these diets have been associated with a lower incidence of cardiovascular diseases, certain types of cancer, and type 2 diabetes than omnivorous diets [68].

At the nutritional level, VEG can be adequate and complete if they are well planned. It is essential to ensure adequate intake of energy, high-quality protein, iron, zinc, calcium, vitamin B12, vitamin D, and omega-3 fatty acids, which may be compromised by these dietary patterns [75].

Common misconceptions suggest that plant proteins are incomplete; however, plant-based foods contain all essential amino acids, and by combining different sources (legumes, grains, nuts), it is possible to achieve a complete protein profile throughout the day [1,75].

#### 2.5.2. Impact on Athletic Performance

The effect of VEG on athletic performance has been a subject of debate. One of the main concerns is the low intake of creatine, carnosine, and certain critical nutrients related to strength and muscle power. However, current scientific evidence indicates that, when properly planned, these diets do not negatively affect athletic performance and may even offer benefits in endurance sports [15,69,70].

##### Endurance Sports

In endurance sports, some studies have suggested that vegetarian diets can improve aerobic capacity by increasing the consumption of antioxidants and complex CHO and lowering energy density, which facilitates weight and body composition control [15]. Additionally, a higher intake of polyphenols and other bioactive compounds may reduce oxidative damage and exercise-induced inflammation [11].

##### Strength and Power Sports

In strength and power sports, the main challenge is to ensure adequate total protein intake and sufficient leucine levels to stimulate muscle protein synthesis. Although the leucine content is usually lower in plant-based sources, strategies such as increasing the total amount of protein or using supplements (soy, pea, or rice protein) can compensate for this difference [66,71,72,75].

##### Team Sports

Evidence regarding team sports is scarce, although no significant differences in performance have been reported between vegetarian and omnivorous athletes [69]. Proper planning is key to ensuring sufficient energy supply and preventing muscle mass loss during long seasons or periods of highly competitive load.

##### Sprint Sports

Sprint performance is sensitive to creatine, carnosine, and leucine availability—nutrients that are typically lower in VEG patterns. Inadequate intake may reduce buffering capacity and repeated sprint ability [66,71,72]. However, with optimized protein distribution and appropriate creatine and beta-alanine supplementation, sprint athletes following VEG can maintain performance. Ensuring sufficient total energy intake is critical to avoid LEA during high-intensity phases.

##### Aesthetic Sports

VEG may offer high micronutrient density and antioxidant benefits, but the risk of LEA, iron deficiency, and suboptimal protein intake is notable in aesthetic athletes [68,71,75]. Careful planning is essential to maintain bone health, hormonal function, and motor coordination—all critical in gymnastics and figure skating [96,97,98]. Supplementation with vitamin B12, vitamin D, calcium, and omega-3 is often necessary.

##### Weight-Category Sports

VEG can support weight regulation because of their high fiber and low energy density, but athletes must avoid excessive restriction, which increases LEA and decreases power output [70,71,75]. Ensuring adequate protein, iron, and zinc intake is essential to preserve strength during weight-cut phases. Creatine supplementation is particularly beneficial in vegan and vegetarian fighters and rowers [66].

#### 2.5.3. Diet-Specific Limitations

Most studies on vegetarian diets and athletic performance have methodological limitations such as small sample sizes and heterogeneous designs. Furthermore, total protein intake and supplement use were not always controlled, which could have significantly influenced the results [15,70].

Likewise, there is significant individual variability in the response to this type of diet, which depends on the discipline practiced, level of competition, the athlete’s nutritional status, and prior experience.

#### 2.5.4. Practical Recommendations

Proper planning is essential for VEG to meet athletes’ nutritional needs. A key aspect is ensuring sufficient energy intake to avoid deficits that could compromise recovery and performance [1]. Because of the higher fiber density and lower energy density of plant-based foods, athletes may experience early satiety, which makes it difficult to achieve the necessary calorie intake [71].

##### Proteins

In vegetarian athletes, protein intake should be at the upper end of the general recommendations for athletes (1.6–2.0 g/kg/day) to compensate for the lower digestibility and protein density of plant-based foods. The distribution of proteins throughout the day and inclusion of leucine-rich sources are key strategies for optimizing protein synthesis [110].

##### Omega-3 Fatty Acids

Such as alpha-linolenic acid (ALA), found in chia seeds, flaxseed, and walnuts, can be converted into eicosapentaenoic acid (EPA) and docosahexaenoic acid (DHA), although this conversion is limited. Microalgae-based supplements can be considered to ensure adequate intake of these fatty acids [111].

##### Vitamin B12

Vitamin B12 is critical and should be supplemented by everyone who follows VEG. Deficiency can have serious consequences for health and performance; therefore, it is advisable to monitor its levels [66,75].

##### Vitamin D and Calcium

Vitamin D is often insufficient in vegan athletes, especially during winter or in indoor sports. It is recommended to assess supplementation on an individual basis and ensure adequate intake of calcium-rich plant-based sources [73,74].

##### Iron and Zinc

Non-heme iron from plant sources has lower bioavailability. Therefore, it is recommended to include a variety of iron-rich foods and improve their absorption by combining them with sources of vitamin C while avoiding phytates, tannins, and calcium in the same meal. Similarly, the limited bioavailability of zinc makes it necessary to incorporate foods rich in zinc and use cooking techniques that enhance its absorption [75].

##### Iodine

Iodine is another critical nutrient, especially for people following a vegan diet. In general, the use of iodized salt is sufficient to meet daily requirements. It should be noted that excessive iodine intake from seaweeds can be harmful [72,75].

##### Specific Supplements

Creatine and beta-alanine, found almost exclusively in animal products, might be of particular interest to vegetarian and vegan athletes who practice strength or power sports. Supplementing with them can help improve anaerobic capacity and recovery [66].

##### Monitoring and Control

It is essential to regularly monitor nutritional status through blood tests and body composition assessments. Planning and support by a specialized dietitian-nutritionist are fundamental to ensuring the success of this type of dietary strategy.

### 2.6. Paleolithic Diets (PALEO)

#### 2.6.1. General Description and Scientific Basis

PALEO is based on the evolutionary hypothesis that the optimal diet for modern humans should resemble that of our Paleolithic hunter-gatherer ancestors. The central premise holds that the human genome has changed very little since the end of the Paleolithic era and that, thereby, we are not fully adapted to the dietary changes brought about by the agricultural revolution approximately 10,000 years ago. This evolutionary discordance has been linked to an increase in chronic diseases such as obesity, type 2 diabetes, and metabolic syndrome [77,78].

Although there was no single PALEO, common patterns can be identified among different hunter-gatherer populations: high consumption of unprocessed foods such as lean meats, fish, fruits, vegetables, nuts, and tubers, with the systematic exclusion of grains, legumes, dairy, salt, added sugars, and ultra-processed products [79].

The approximate macronutrient distribution of PALEO includes 19–35% of energy from animal proteins, 28–58% from fats (predominantly unsaturated with a high proportion of omega-3), and 22–40% from complex CHO derived from fruits, roots, and vegetables. The diet is also characterized by high nutritional density, low glycemic index, high content of both soluble and insoluble fibers, and high concentrations of vitamins, minerals, phytochemicals, and antioxidants [80].

From a historical and anthropological perspective, studies such as Murdock’s Ethnographic Atlas (1967) [112] and Wrangham’s work [113] have helped strengthen the hypothesis that the consumption of cooked and nutrient-dense foods is key to human evolution. The diet was popularized in scientific and popular literature by authors such as Loren Cordain, gaining widespread acceptance in fitness and CrossFit^®^ circles, although it is followed less widely today [114].

#### 2.6.2. Impact on Athletic Performance

##### Endurance Sports

PALEO presents significant limitations for endurance sports owing to its low CHO content, which compromises glycogen resynthesis and reduces the availability of energy substrates during prolonged exercise [81]. Although it has been suggested that their high nutritional density and antioxidant content could promote certain mitochondrial adaptations, the evidence indicates that excessive CHO restriction may impair performance in events lasting more than 60–90 min [44]

Some nutritional periodization models may integrate periods of low CHO availability to promote metabolic efficiency, but rigidly applying this strategy throughout the season does not appear to be compatible with maintaining performance in endurance sports [115].

##### Strength and Power Sports

In these disciplines, PALEO could offer benefits in terms of body composition and muscle recovery owing to its high protein content and healthy fats [85,114]. However, the low availability of CHO can limit performance in repeated high-intensity efforts and rapid replenishment of glycogen post-training, especially in routines with high frequency or volume.

Some theoretical studies have suggested that this diet may be useful during the cutting phases or for body weight control, but its ergogenic impact on strength athletes is still poorly documented in well-controlled trials [115].

##### Team Sports

Team sports have a high glycolytic demand owing to a combination of intermittent efforts. A study conducted by Pięta et al. (2023) on professional handball players subjected to a PALEO intervention for 8 weeks showed a significant reduction in body mass and improvements in some metabolic markers, such as adiponectin [82].

However, a similar study observed a decrease in total work performed and in average and maximum anaerobic power, suggesting that PALEO may not be optimal in sports contexts that require high glycogen availability to maintain explosive actions and rapid recovery between efforts [83].

##### Sprint Sports

The relatively low CHO availability characteristic of PALEO may reduce glycolytic capacity and impair repeated sprint performance [81,82,83]. Although PALEO provides high-quality proteins, insufficient CHO supply limits neuromuscular output and rapid phosphocreatine resynthesis. Sprint athletes, therefore, require strategic CHO inclusion around high-intensity sessions if PALEO is used.

##### Aesthetic Sports

PALEO’s exclusion of grains and dairy can increase the risk of deficiencies in calcium, vitamin D, and CHO—nutrients essential for bone health, hormonal function, and high-quality technical training [95,98,116,117]. The restrictive nature of this diet may exacerbate LEA risk in athletes who are already vulnerable to RED-S. PALEO thus requires careful adaptation to ensure adequate energy and micronutrient intake in aesthetic sports.

##### Weight-Category Sports

PALEO may assist with short-term reductions in fat mass while maintaining lean body mass; however, limited CHO intake can impair power, repeated-effort capacity, and hydration management, which are crucial in combat sports [82,83]. Its restrictive profile may also increase LEA and micronutrient deficiency risk if applied during weight-cut periods. Strategic CHO intake before, during, and/or immediately after training becomes essential when a PALEO diet is employed in weight-category sports.

#### 2.6.3. Diet-Specific Limitations

The scientific literature on the application of PALEO in sports has several limitations. Many studies have been conducted on sedentary or recreational subjects with short interventions and small sample sizes, making it difficult to extrapolate the results to trained athletes [83,84].

In addition, there is significant heterogeneity among the versions of PALEO used in research, some with a higher CHO content (up to 35–40%) and others closer to ketosis (<10% CHO), which prevents a uniform comparative analysis [76].

The exclusion of entire food groups, such as dairy and legumes, poses a risk of nutritional deficiencies in the medium- and long-term. In particular, a low intake of calcium and vitamin D can compromise bone health, especially in female athletes at risk of low energy availability and hormonal imbalances [118].

It has also been noted that the restrictive nature of PALEO can decrease long-term adherence and lead to an excessively rigid approach in athletes who need flexibility to respond to the different phases of training and competition [85,86].

#### 2.6.4. Practical Recommendations

The implementation of PALEO in sports should be individualized and guided by qualified professionals. For endurance or team sports, its use may be considered during specific phases of training, but always with strategic adjustments to include energy-dense CHO sources (such as potatoes, sweet potatoes, dried fruits, or honey), especially before key sessions.

A high protein intake can support adaptations in strength and power sports; however, incorporating targeted CHO intake around training sessions may be essential to optimize performance and recovery [95].

Special attention should be paid to bone and hormonal health, total energy sufficiency, and micronutrient profile of the diet. PALEO can form part of a nutritional periodization strategy, but its prolonged and exclusive use during high-intensity or competitive phases is not recommended. Finally, a flexible approach is suggested that gradually incorporates certain strategic foods according to individual tolerance, sporting context, and specific objectives to avoid nutritional deficiencies, improve adherence, and ensure long-term safety.

### 2.7. Carbohydrate Periodization (CP)

#### 2.7.1. General Overview and Scientific Foundations

CP is an advanced nutritional strategy that involves adapting CHO intake in a planned and strategic manner throughout the training and competition cycles. Its goal is to optimize metabolic adaptations to training, improve performance, and facilitate recovery [9].

Traditionally, a high and consistent intake of CHO has been recommended to maintain elevated glycogen stores and support performance during intense or prolonged sessions. However, recent studies have shown that training under low CHO availability can promote favorable adaptations, such as increased mitochondrial biogenesis, enhanced oxidative capacity, and improvements in metabolic flexibility [18,90].

The physiological basis focuses on manipulating glycogen stores and the exogenous availability of CHO to modulate signaling pathways such as AMPK, p38 MAPK, and PGC-1α, which are involved in improving energy efficiency and aerobic capacity [27].

Common CP methods include:

- “Train low, compete high”: train with low CHO reserves to stimulate adaptations, but compete with full reserves to maximize performance [6,88].
- “Sleep low”: perform an evening session at high intensity without subsequent CHO replenishment, sleeping with reduced glycogen, followed by a fasted session the next day [87].
- Double training session: two sessions on the same day, consuming little CHO between them, to train the second session with low reserves [119].

These approaches should be applied carefully and individually, as an excessive or prolonged reduction in CHO can compromise training quality and increase the risk of illness or injury [119].

#### 2.7.2. Impact on Athletic Performance

##### Endurance Sports

Evidence suggests that CP can enhance metabolic adaptations in endurance sports, such as an increased capacity to oxidize fats and preserve glycogen during prolonged effort [12]. These adaptations could delay fatigue during ultra-endurance events or prolonged tests (>2–3 h).

However, this does not always translate into direct improvements in competitive performance, especially in events that require maintaining intensities close to the anaerobic threshold, where CHO availability remains a determining factor [13]. Therefore, it is recommended to alternate “low” and “high” sessions according to the objective of the training cycle and the type of session.

##### Strength and Power Sports

In sports based on power and explosive strength, CHO are essential for maintaining the ability to perform repeated maximal effort. Although training with low CHO availability may induce certain metabolic adaptations, CHO reduction is not recommended in these disciplines, as it could compromise the quality of the session and strength gains [95].

##### Team Sports

In team sports (soccer, basketball, and hockey), the ability to perform repeated sprints and maintain intensity is highly dependent on muscle glycogen. CP may be used during preparatory phases to improve aerobic capacity and fat oxidation, during competitive and high-load phases, and maintaining a high CHO intake is a priority to sustain performance [1].

##### Sprint Sports

Sprint athletes require frequent high-CHO availability to sustain maximal neuromuscular output. Periodized “train-low” sessions may enhance metabolic signaling but should be minimized in sprinters, because low-CHO training reduces power, technique quality, and session intensity [9,119]. CP can be applied selectively, but “compete high” and “sprint-session high” are essential principles for this group.

##### Aesthetic Sports

Aesthetic athletes benefit from CP because it allows technical training to occur with adequate CHO availability while enabling tighter energy control during lighter sessions. This approach helps reduce LEA risk and supports technical skill acquisition, concentration, and hormonal balance during demanding practices [98,116,117]. Strategic high-CHO days before choreography or apparatus sessions improve precision and may reduce injury risk.

##### Weight-Category Sports

CP is particularly useful in weight-category sports because it allows controlled CHO reduction during early preparatory phases while preserving glycogen availability before sparring simulations and competition [9,18,27]. Alternating low- and high-CHO days helps regulate body mass without compromising strength and speed. Reintroduction of high-CHO availability before weigh-ins and competition is essential for optimal performance.

#### 2.7.3. Diet-Specific Limitations

CP is a complex strategy that requires a high degree of planning. Its inadequate application can lead to low-quality training, increased fatigue, decreased immune function, and a higher risk of injury [120].

There were marked individual differences in the capacity to adapt to training with low CHO availability. Additionally, the literature presents heterogeneous results, with differences in the duration of the protocols, the magnitude of CHO restriction, and the profiles of the participants [45].

#### 2.7.4. Practical Recommendations

The implementation of CP should be based on a detailed assessment of the sports discipline, competition calendar, and the athlete’s level.

In endurance sports, the “train low” strategy can be used in low- or moderate-intensity sessions aimed at promoting metabolic adaptations, while in key sessions or competitions, high CHO availability (“compete high”) should be prioritized to optimize performance [18,88].

In strength and team sports, CHO reduction should be used with caution and only in controlled contexts, such as base phases, where the goal is to improve general aerobic capacity. In sessions focused on power, hypertrophy, or explosiveness, it is essential to maintain a high CHO availability [1,119].

It is essential to ensure an adequate protein intake during periods of low CHO availability to minimize the loss of lean mass and promote muscle recovery [110]. In addition, key micronutrients and proper hydration must be ensured, especially during training periods in the heat or under extreme environmental conditions. CHO periodization should be planned by a sports nutritionist and monitored by parameters such as body composition, recovery capacity, sleep quality, and overall health.

Similarly, it is advisable to adapt the strategy according to the athlete’s individual response and tolerance. Lastly, constant communication between the technical team and the nutritionist is recommended to adjust CHO strategies to the needs of each training and competition block, always keeping health as the main priority.

To operationalize the third objective of this consensus, Table 2 summarizes the applicability of each dietary strategy across different sport categories. This overview considers the specific physiological demands of endurance, strength–power, sprint, team, weight-category, and aesthetic sports, supporting evidence-based decision-making in applied settings.

## 3. Key Factors in the Application of Dietary Strategies

The implementation of a dietary strategy in athletes does not depend solely on the selection of an eating pattern or the manipulation of macronutrients but rather requires a comprehensive and multidimensional analysis. This individualization is essential for optimizing performance, ensuring safety, and promoting long-term health [1,3].

### 3.1. Individual Needs and Biological Variability

Each athlete exhibits a unique metabolic and physiological response to different types of diet, determined by genetic, epigenetic, sex, age, body composition, and training status factors [121]. Genetics can influence the efficiency of energy metabolism, tolerance to certain nutrients, and response to energy restriction or excess [96].

Additionally, variables such as circadian rhythm, chrononutrition, and personal or cultural preferences play a fundamental role in the adherence to and effectiveness of the strategy [97].

### 3.2. Type of Sport and Effort Profile

The choice of dietary strategies should be tailored to the energy and metabolic demands of the sport. Endurance sports require maximizing glycogen stores and CHO availability to sustain prolonged high-intensity efforts, whereas strength and power sports demand a focus on preserving muscle mass and explosive capacity [9].

In team sports, characterized by intermittent high-intensity efforts and brief recovery periods, it is essential to ensure rapid glycogen recovery and optimize hydration [7].

In addition to these traditional categories, other sport groups present specific nutritional needs that do not fully align with the previous classification. Sprint sports rely primarily on maximal anaerobic power and phosphagen system availability, requiring dietary approaches that prioritize glycogen preservation, rapid energy turnover, and neuromuscular function [91,93]. Aesthetic sports, such as gymnastics or figure skating, combine high technical demands with strict body-composition targets, making energy availability, micronutrient adequacy, and psychological safety particularly relevant [98,116]. Weight-category sports (e.g., combat sports, lightweight rowing) require strategic body-mass regulation while maintaining performance, which often necessitates tightly monitored nutrition planning to avoid the risks associated with rapid weight loss [8,118].

Across all sport types, nutrient timing remains essential: the availability of energy substrates before, during, and after activity directly influences performance, metabolic adaptation, and recovery. Consuming carbohydrates post-exercise supports glycogen resynthesis, while combining CHO with high-quality proteins enhances muscle repair and promotes optimal recovery [2].

### 3.3. Health Status and Clinical Situation

The general health status and presence of specific conditions (nutritional deficiencies, gastrointestinal disorders, allergies, or intolerance) must be considered when applying any dietary strategy. Restrictive diets or those with limited food groups, such as the KD or PALEO, require strict medical and nutritional supervision to prevent deficiencies and metabolic disturbances [8].

In female athletes, it is especially important to monitor the available energy to prevent the female athlete triad and RED-S syndrome, which negatively affects menstrual function, bone health, and immunity [98].

### 3.4. Nutritional Periodization

Nutritional periodization involves adapting macronutrients and energy intake to training and competition cycles. This dynamic approach promotes specific adaptations during certain phases (e.g., increased fat oxidation during the aerobic base phase or maximization of CHO during high-intensity phases) and optimizes performance at key moments [18].

The combination of training with low and high CHO availability (“train low, compete high” strategy) can stimulate beneficial metabolic adaptations, but it should be applied with caution and not generalized to all athletes [95].

### 3.5. Body Composition and Aesthetic Goals

Many athletes seek to manipulate their body composition to improve their power-to-weight ratio, optimize efficiency, or fit weight categories. In such cases, it is essential to implement dietary strategies that promote fat loss while preserving lean mass, avoid performance loss, and minimize the risk of both physical and psychological injuries [117].

The calorie deficit should be moderate and planned, combining an adequate protein intake (1.6–2.4 g/kg/day) and periodizing energy intake according to training load [110].

### 3.6. Psychological Factors and Adherence

The success of any dietary intervention largely depends on adherence. The perception of rigidity, excessive restrictions, or incompatibility with social life can compromise the long-term sustainability of the strategy [122].

It is crucial for athletes to actively participate in decision-making, understand the reasoning behind each adjustment, and maintain a degree of flexibility to improve their relationship with food and prevent dysfunctional eating behaviors.

### 3.7. Control and Monitoring

Continuous evaluation is essential for adjusting the strategy and ensuring its effectiveness and safety. The use of objective indicators, such as body weight, body composition (DEXA, bioimpedance, or anthropometry), biochemical parameters (i.e., hemoglobin, ferritin, vitamin D, and lipid profile), and the assessment of sports performance (specific tests and competition data) are recommended [99]. It is important to consider that exercise produces changes in biochemical and hematological basal values of athletes compared to the general population [123].

In addition, subjective aspects such as perceived fatigue, sleep quality, mood, and overall well-being should be monitored, integrating a comprehensive view of the athlete’s condition.

### 3.8. Ethical Considerations and Sustainability

Ethical, cultural, and environmental considerations increasingly influence athletes’ dietary choices and should be integrated into individualized nutrition planning [65,66,71,75]. Many athletes express interest in VEG or predominantly plant-based eating patterns due to ethical, environmental, or health-related motivations [66,70,71,73,74,75]. However, sustainability is a multidimensional construct that depends on multiple factors—production systems, food processing, geographic origin, seasonality, and waste management, among others—and not all VEG are inherently more sustainable, nor do all omnivorous diets have the same environmental impact [65,67,75,124].

From a performance and health perspective, VEG can be compatible with high-level sport when appropriately planned, but they may require close monitoring of critical nutrients such as iron, vitamin B12, calcium, zinc, omega-3 fatty acids, and protein quality [66,70,71,73,74,75]. At the same time, athletes who choose mixed or omnivorous diets may also adopt sustainable and ethical practices through the selection of minimally processed foods, seasonal produce, and responsibly sourced animal products [65,75].

Sports nutrition professionals should therefore provide evidence-based guidance that respects the athlete’s values while ensuring nutritional adequacy, minimizing unnecessary restrictions, and maintaining performance [1,3,8,66,71]. This includes evaluating the environmental implications of different dietary strategies within a broader systems perspective, avoiding simplified assumptions, and promoting balanced, flexible, and culturally appropriate approaches [65,67,70,75,124].

### 3.9. Sex-Specific Considerations in Female Athletes

Sex-specific physiological characteristics require tailored attention when implementing nutritional strategies in female athletes. Hormonal fluctuations across the menstrual cycle, as well as the use of hormonal contraceptives, influence substrate metabolism, recovery capacity, thermoregulation, and injury risk [98,116,117]. Compared with men, female athletes exhibit a greater reliance on fat oxidation at submaximal intensities and may therefore require targeted carbohydrate adjustments around high-intensity training blocks to ensure adequate glycogen availability [98,122].

A central concern in this population is the risk of LEA and the associated clinical spectrum that includes menstrual dysfunction, impaired bone turnover, and the broader framework of RED-S. These conditions can compromise performance, recovery, immune competence, and long-term health, particularly in aesthetic disciplines, endurance sports, and weight-category events [98,116,117,125].

Nutritional strategies for female athletes should prioritize adequate total energy intake, optimal protein distribution throughout the day (including leucine-rich sources), and appropriate micronutrient status—especially iron, calcium, vitamin D, and omega-3 fatty acids. Carbohydrate intake may require specific adaptation during the late luteal phase because of the increased metabolic cost and thermogenic changes observed at this stage, while adequate post-exercise fueling is essential to mitigate training-induced hormonal fluctuations [98,125].

Regular monitoring of menstrual function, iron status, bone health, and symptoms related to RED-S is recommended to guide individualisation across the season. When integrated with the sport-specific considerations detailed throughout this consensus, these principles provide a comprehensive framework for safe and effective nutrition planning in female athletes.

## 4. Controversies and Limitations

Although the diet-specific limitations for each nutritional approach are addressed within their respective sections in Section 2, broader controversies and cross-cutting methodological challenges remain. The following chapter synthesizes the overarching limitations that affect the interpretation, generalizability, and application of sports nutrition research across all dietary strategies.

Sports nutrition is a constantly evolving field that integrates multiple dietary strategies to improve athletic performance, body composition, and health. However, the practical application of these strategies is subject to numerous controversies and limitations stemming from both the individual variability and methodological quality of the available studies.

### 4.1. Heterogeneity in Studies and Limited Evidence

One of the main limitations of the current scientific literature is the significant heterogeneity among studies, which makes direct comparison between results difficult. Differences in experimental design, sample size, duration of interventions, type of athletes (elite vs. recreational), sports disciplines, and methods for evaluating performance all contribute to inconsistent results [1,5].

Furthermore, many studies have used acute or short-term models that do not accurately reflect the chronic effects of nutritional strategies applied in the real-world context of training and competition [9]. Most studies have also been conducted under controlled laboratory conditions, which may limit their applicability in field situations, where factors such as competitive stress, environmental conditions, or variability in food access come into play [117].

### 4.2. Inter-Individual Variability

The response to nutritional strategies shows high inter-individual variability and is influenced by genetic factors, sex, age, training level, gut microbiota, and hormonal status [2,99]. This variability complicates the formulation of universal recommendations and highlights the importance of individualization.

For example, in response to LCD or KD, some athletes experience improvements in fat oxidation and weight control, whereas others exhibit significant reductions in anaerobic performance and the ability to perform high-intensity efforts [12]. The same applies to IF, where adaptation and tolerance vary widely among individuals [52].

### 4.3. Potential Risks and Nutritional Deficiencies

Restrictive strategies, such as the KD, strict vegetarian diets, or the PALEO, may carry risks of nutritional deficiencies if not properly planned. Among the critical nutrients are iron, calcium, vitamin B12, vitamin D, zinc, and omega-3 fatty acids [8].

In athletes, these deficiencies can negatively affect bone health, the immune system, muscle recovery, and cognitive function, as well as increase the risk of injuries and chronic fatigue [98].

In women, the risk of deficiencies is even higher because of the greater demand for iron and calcium and the susceptibility to developing RED-S, which can affect menstrual function, bone health, and metabolic health [116].

### 4.4. Influence of External and Social Factors

Adherence to specific dietary strategies is influenced by social, cultural, economic, and psychological factors. Social pressure, food availability, and environmental support are key elements in the success or failure of interventions [126].

Additionally, the spread of biased or non-evidence-based information on social networks and digital media contributes to confusion, promotes extreme diets, and fosters unrealistic expectations among athletes and coaches [124]. The growing popularity of “influencers” and sports nutrition gurus has amplified the adoption of strategies without critical evaluation, thereby increasing the risk of harmful practices.

### 4.5. Controversies Surrounding Timing and Periodization

Although the concept of “nutrient timing” and CHO periodization has gained popularity, the evidence regarding their specific benefits remains a matter of debate. While there is support for post-exercise protein consumption to maximize protein synthesis and recovery, precise “timing” does not always translate into significant long-term performance benefits [94].

Similarly, although training with low CHO availability can promote metabolic adaptations, it does not always result in direct performance improvements and could compromise the intensity and quality of the training [18].

### 4.6. Difficulty in Evaluating Performance

Objectively assessing the impact of nutritional strategy on performance is complex. Athletic performance is determined by multiple factors, including physical training, psychology, techniques, tactics, and external conditions. Isolating the effects of diet is extremely difficult under field conditions [127].

Likewise, the positive results observed in certain studies may not be clinically relevant or may not translate into a significant improvement in actual competition, underscoring the need to interpret the data with caution [100].

### 4.7. Integrative Perspective and Future Outlook

The current controversies and limitations in sports nutrition highlight the importance of adopting a critical, evidence-based, and deeply individualized approach. Collaboration between nutritionists, sports physicians, coaches, and athletes is essential for designing and adjusting safe and effective nutritional strategies.

The science of nutrition applied to sports must move towards higher-quality studies involving larger populations and assessments under real training and competitive conditions. Only in this manner can a solid foundation be provided to guide practice and reduce the gap between theory and real-world applications [3].

## 5. Conclusions and Final Recommendations

This consensus document aimed to offer a critical and up-to-date overview of the main dietary strategies used by athletes across different disciplines. A detailed analysis of each strategy allows for clear conclusions that help guide professional practice and promote evidence-based decision-making.

### 5.1. Individualization as a Key Principle

One of the most consistent findings of the strategies reviewed was the importance of individualization. The response to diet is highly variable and depends on factors such as genetics, sex, age, type of sport, phase of the season, and personal and cultural preferences [1].

Each strategy may offer potential benefits in certain contexts, but none are universally superior or broadly applicable to all athletes. The success of any dietary intervention depends on personalization and continuous adaptation, tailored to the training and competition cycle [9].

### 5.2. Importance of Adequate Carbohydrate Intake

CHO remains a central macronutrient for athletic performance, particularly in endurance sports, team sports, and high-intensity disciplines. Evidence supports that adequate CHO availability optimizes glycogen resynthesis, maintains exercise intensity, and facilitates recovery [5].

The CHO periodization and the strategic use of low-availability training (“train low”) can promote metabolic adaptations, but should be applied with caution and in specific phases of the training plan to avoid compromising performance or immune function [18].

### 5.3. Role of Low-Carbohydrate and Ketogenic Diets

LCD and KD have been shown to increase fat oxidation and improve body composition in certain cases. However, their use should be selective, especially in disciplines with a high glycolytic demand or those that require repeated high-intensity efforts [46].

In ultra-endurance sports or preparatory phases, in which promoting metabolic flexibility is the goal, they can play a useful role. However, reducing CHO levels does not always lead to direct improvements in competitive performance, and prolonged CHO use may affect training quality [12].

### 5.4. Intermittent Fasting and Time-Restricted Eating

IF is an effective strategy to improve body composition and metabolic parameters. However, the evidence in athletes is limited and heterogeneous. Its implementation should be supervised and scheduled when the energy availability required for key sessions is not compromised [52].

IF can be useful during periods of fat loss; however, it is essential to ensure the preservation of lean mass through adequate protein intake and careful meal distribution [110].

### 5.5. Vegetarian and Vegan Diets

VEG can support athletic performance if they are properly planned. Ensuring adequate intake of energy, protein, iron, calcium, vitamin B12, and omega-3 is essential to avoid deficiencies that could compromise health and performance [128,129].

Growing interest in these patterns is associated with ethical and environmental concerns. Therefore, healthcare professionals should offer complete and safe nutritional plans [100].

### 5.6. Paleolithic Diet

The PALEO provides a high content of protein and micronutrients, but its low supply of CHO can limit endurance and team sports. Its use could be considered during specific periods of the season to promote metabolic adaptations, although careful planning is required to avoid deficiencies and performance losses [20].

### 5.7. Role of Supervision and Monitoring

Continuous monitoring and nutritional education are essential to ensure the effectiveness and safety of dietary strategies. It is recommended to track objective markers (body composition, biochemical parameters, and performance) and subjective markers (feelings of fatigue and general well-being) to adjust the nutritional plan dynamically [99].

Collaboration between nutritionists, trainers, sports doctors, and athletes is key to achieving a comprehensive approach and ensuring that nutrition is harmoniously integrated with training and individual goals [3,7].

### 5.8. Final Considerations and Future Lines of Research

Available evidence shows that there is no “ideal” diet that is superior for all athletes and situations. Practical applications should be guided by the principles of individualization, flexibility, and sustainability.

In clinical and sports practice, decisions must be based on current scientific evidence, always respecting athletes’ preferences and context. It is essential to avoid dogmatic or trend-based recommendations and prioritize a critical and adaptive approach.

Future research should focus on the following aspects:

- Longitudinal studies in real training and competition conditions.
- Evaluating the long-term impact of specific strategies on health and performance.
- Exploring the role of genetics and gut microbiota in the dietary response.
- Analysis of the interaction between nutrition, chronobiology, and circadian rhythm.
- Assessing sustainable and ethical dietary strategies in high-performance athletes.

Advances in these areas will make it possible to offer more personalized and robust evidence-based recommendations, promoting athletic performance, as well as the holistic health and well-being of athletes.

## Figures and Tables

**Table 2:** Applicability of dietary strategies across different sport categories.

Strategy	Endurance Sports	Strength/Power Sports	Team Sports	Sprint Sports	Weight-Category Sports	Aesthetic Sports
**HCD**	Highly recommended; maximizes glycogen and supports prolonged high-intensity effort.	Useful around high-volume training days; less critical in maximal efforts.	Recommended for match days and congested schedules requiring repeated sprints.	Supports repeated sprint ability; useful pre-competition.	Requires careful energy control; may increase weight if poorly periodized.	Appropriate when portion-controlled; risk of energy surplus if misapplied.
**LCD**	Useful for base training and enhancing fat oxidation; not suitable for competition phases.	Limited benefit; may impair high-intensity or explosive output.	Not recommended due to the metabolic cost of accelerations and sprints.	Generally not compatible with maximal speed requirements.	May assist short-term weight reduction; monitor fatigue.	Can reduce training quality and increase perceived exertion.
**LCHF**	Potential benefit in ultra-endurance; reduces reliance on CHO during long events.	Not recommended; compromises anaerobic and phosphagen systems.	Poor fit due to intermittent high-intensity demands.	Contraindicated for pure sprinters.	May assist weight-cutting phases; risk of performance decline.	Not suitable due to the need for high intensity, jump, and power outputs.
**KD**	Effective for extreme endurance or body-mass reduction phases; not recommended near competition.	Markedly reduces power output; not advised.	Not suitable; reduces high-intensity performance.	Contraindicated; impairs peak power and sprint speed.	May be used short-term for rapid weight reduction with supervision.	Increases fatigue; may reduce lean mass; not advisable.
**IF**	Possible use during low-intensity phases; monitor energy availability.	Risk of reduced strength gains and impaired recovery.	May compromise performance in afternoon/evening sessions with long fasting windows.	Not advised; low energy availability impairs neuromuscular performance.	Can be considered for short weight-cut periods under professional oversight.	Risk of inadequate energy availability and RED-S; caution required.
**VEG**	Suitable with adequate energy and nutrient planning; supports cardiovascular health.	Compatible with strength sports, provided protein quality and iron/B12 are monitored.	Appropriate if energy intake meets training demands.	Viable with optimized protein timing; creatine/beta-alanine supplementation recommended.	Suitable for individualized planning; attention to protein density.	Common choice; requires monitoring of iron, B12, and omega-3.
**PALEO**	Can improve glycemic control and body composition; CHO intake may be insufficient for high-intensity events.	Acceptable in off-season phases; may limit glycogen availability for power training.	Not recommended in season due to low CHO availability.	Typically inadequate for sprint fueling.	Useful for fat reduction phases; ensure calcium/vitamin D intake.	May assist leanness goals but risks nutrient deficiencies.
**CP**	Strong evidence for enhancing metabolic adaptations; “broadly applicable across sport categories.	Useful for optimizing training stimuli; maintain high CHO on explosive days.	Ideal for alternating heavy and light training loads.	Supports glycogen optimization around sprint sessions.	Allows flexible fueling while maintaining weight targets.	Suitable if energy availability is protected.

Abbreviations: HCD: high-carbohydrate diet; LCD: low-carbohydrate diet; LCHF: low-carbohydrate high-fat diet; KD: ketogenic diet; IF: intermittent fasting; VEG: vegetarian/vegan diet; PALEO: Paleolithic diet; CP: carbohydrate periodization. Note: Female athletes require additional consideration of energy availability, iron status, menstrual function, and RED-S risk across all dietary strategies (see Section 3). Evidence synthesis: This table summarizes the sport-specific applicability discussed in Section 2 and Section 3 and does not introduce evidence beyond what is referenced in the main manuscript.
